# Notch Signaling in Vascular Endothelial Cells, Angiogenesis, and Tumor Progression: An Update and Prospective

**DOI:** 10.3389/fcell.2021.642352

**Published:** 2021-02-16

**Authors:** Abdellah Akil, Ana K. Gutiérrez-García, Rachael Guenter, J. Bart Rose, Adam W. Beck, Herbert Chen, Bin Ren

**Affiliations:** ^1^Department of Surgery, School of Medicine, University of Alabama at Birmingham, Birmingham, AL, United States; ^2^O’Neal Comprehensive Cancer Center, School of Medicine, University of Alabama at Birmingham, Birmingham, AL, United States

**Keywords:** angiogenesis, arteriolar niche, cancer stem cell, Notch signaling, tumor microenvironment

## Abstract

The Notch signaling pathway plays an essential role in a wide variety of biological processes including cell fate determination of vascular endothelial cells and the regulation of arterial differentiation and angiogenesis. The Notch pathway is also an essential regulator of tumor growth and survival by functioning as either an oncogene or a tumor suppressor in a context-dependent manner. Crosstalk between the Notch and other signaling pathways is also pivotal in tumor progression by promoting cancer cell growth, migration, invasion, metastasis, tumor angiogenesis, and the expansion of cancer stem cells (CSCs). In this review, we provide an overview and update of Notch signaling in endothelial cell fate determination and functioning, angiogenesis, and tumor progression, particularly in the development of CSCs and therapeutic resistance. We further summarize recent studies on how endothelial signaling crosstalk with the Notch pathway contributes to tumor angiogenesis and the development of CSCs, thereby providing insights into vascular biology within the tumor microenvironment and tumor progression.

## Overview of the Notch Signaling Pathway

The Notch signaling pathway is highly conserved across all vertebrate species and orchestrates a diverse range of functions including cell growth, differentiation, and patterning. In mammals, this pathway features four transmembrane receptors (Notch 1–4) that interact with five canonical transmembrane ligands (Jagged 1, 2; Delta-like ligand 1, 3, and 4) (Aquila et al., 2019). Canonical Notch signaling has been more widely characterized than non-canonical Notch signaling. Activation of the canonical Notch pathway begins when a Notch receptor extracellularly interacts with a Notch ligand, which subsequently initiates proteolytic cleavage of the receptor. This cleavage precedes two sequential proteolytic events that require the enzymes including the disintegrin metalloproteinase domain-containing protein 10 (ADAM10) and the γ-secretase. The series of cleavage events following the initial Notch receptor–ligand interaction result in the release of the Notch receptor intracellular domain (NICD) from the cell membrane. The NICD then translocates to the nucleus where it acts as a transcriptional coactivator in cooperation with recombination of signal-binding protein for the immunoglobulin kappa J region (RBPJ).

**FIGURE 1:**
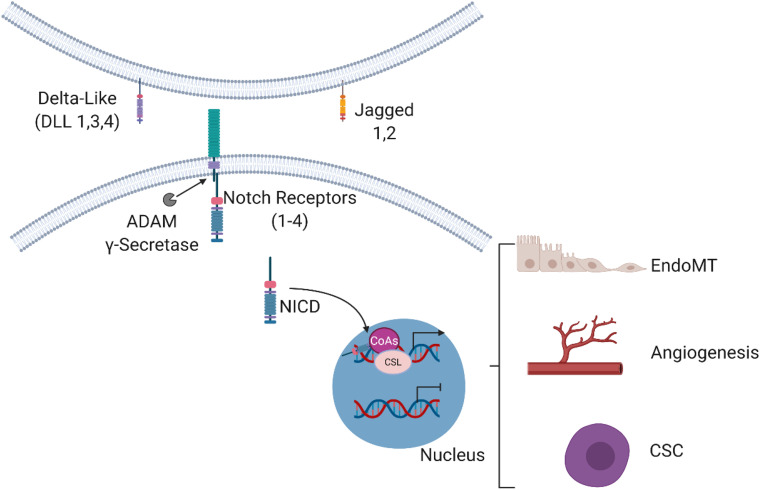
Overview of the Notch signaling pathway. Binding of the Notch ligands (Delta-like 1, Delta-like 3, Delta-like 4, Jagged 1 and Jagged 2) to the transmembrane Notch receptors (Notch 1–4) results in proteolytic cleavage events by ADAM and γ-secretase to mediate the release of the Notch intracellular domain (NICD). The NICD then enters the nucleus and interacts with cofactors to regulate the expression of target genes involved in EndoMT, angiogenesis, and CSC biology.

The NICD–RBPJ interaction initiates transcription of Notch target genes to regulate cell fate determination, migration, differentiation, and proliferation under both normal and malignant conditions (Figure 1). Consequently, Notch signaling can control the expression of a group of target genes that ultimately impact critical cellular functions (D’Souza et al., 2010; Kangsamaksin et al., 2014).

In contrast, non-canonical Notch signaling can be initiated by a non-canonical ligand and may not require cleavage of the Notch receptors. In some forms of non-canonical signaling, there is no involvement of RBPJ, which may reflect interactions with other signaling pathways upstream of the NICD–RBPJ complex (Andersson et al., 2011). Given the diverse roles of Notch signaling in different contexts, it is not surprising that defects in Notch ligands or perturbations in the overall Notch pathway have been associated with genetic disorders and cancers.

## Notch Signaling in Endothelial Cells and Angiogenesis

Angiogenesis in general refers to the formation of new blood vessels from preexisting vasculature, a process that is vital for normal tissue growth, development, and wound healing (Ren et al., 2019; Liu et al., 2020). Blood vessels make up complex vascular networks composed of arteries (arteriogenesis and *de novo* arteriogenesis), capillaries (vasculogenesis), and veins (venogenesis). These networks supply oxygen and nutrients to all tissues and remove wastes in the body in support of tissue growth and organ function under physiological and pathological conditions (Sitohy et al., 2011; Nagy and Dvorak, 2012; Ren et al., 2019). The process of generating new blood vessels occurs through several different mechanisms. One mechanism is the sprouting angiogenesis. This process is initiated by the release of growth factors from cells and requires the coordination of a series of cellular processes. One key process is the selection of a migrating tip cell at the end of the sprout, a tightly coordinated balance between newly forming endothelial cells (ECs), and the maintenance of existing vascular tubes (Otrock et al., 2007; Blanco and Gerhardt, 2013).

Studies suggest that Notch signaling plays an essential role in angiogenesis through interactions with the Notch ligands, in addition to crosstalk with other pathways such as vascular endothelial growth factor (VEGF) signaling. Delta-like ligand 4 (DLL4) has been shown to be a critical Notch ligand for stimulating angiogenesis (Benedito et al., 2009; Blanco and Gerhardt, 2013; Oon et al., 2017; Naito et al., 2020). However, another Notch ligand, Jagged1 (Jag1), can compete with DLL4 to negatively regulate angiogenesis (Taslimi and Das, 2018). This concept was recently illustrated in an *in vitro* study showing that Jag1 is a potent pro-angiogenic regulator capable of antagonizing DLL4–mediated Notch signaling in angiogenesis (Benedito et al., 2009).

Moreover, Notch signaling may directly and/or indirectly regulate angiogenesis through crosstalk with VEGF receptors (VEFGRs) (Garcia and Kandel, 2012; Luo et al., 2019). For instance, in a murine retinal model, DLL4 may act downstream of VEGF as a negative regulator of VEGF-mediated angiogenesis (Suchting et al., 2007). Notch signaling can therefore influence both the induction and the cessation of angiogenesis through different mechanisms. This apparent involvement of Notch signaling emphasizes its potential role as a target for controlling angiogenesis.

The ability of Notch signaling to dictate angiogenetic processes becomes important in the context of aberrant angiogenesis. Pathological angiogenesis plays an important role in abnormal vessel growth and contributes to the pathogenesis of such human diseases as diabetic retinopathy, ischemic cardiovascular diseases, inflammatory processes (rheumatoid arthritis), and cancer (Carmeliet, 2005). In cancer, angiogenesis is critical for disease progression. Abnormal angiogenesis in the tumor microenvironment (TME) can promote tumor growth and progression (Carmeliet, 2005). Therefore, targeting different aspects of the Notch signaling pathway may be a feasible approach to inhibiting pathogenic angiogenesis. For example, therapeutically inhibiting DLL4 in murine ischemic retinas has shown potential to promote the growth of normal blood vessels to partially reverse ischemic conditions (Lobov and Mikhailova, 2018). In addition, blocking the DLL4/Notch1 interaction in cancer is being explored in clinical trials because DLL4 inhibition has been shown to cause non-productive vessel formation within the TME, thereby inhibiting tumor growth (Kuhnert et al., 2011). Designing targeted treatments against Notch signaling may thus demonstrate therapeutic potential by regulating angiogenesis in different disease contexts.

Notch signaling is also implicated in arteriogenesis and *de novo* arteriogenesis, the growth of functional collateral arteries from preexisting arterio-arteriolar anastomoses and *de novo* arterial development, in which smooth muscle cells (SMCs) cover ECs (Heil et al., 2006). The preferential expression of DLL4 on arterial ECs highlights the importance of Notch signaling in arteriogenesis (Shutter et al., 2000; Liu et al., 2003). In fact, DLL4/Notch1 signaling may function downstream of VEGF during arteriogenesis and likely supports the survival and differentiation of arterial ECs (Lawson et al., 2001). However, the complete function of Notch signaling during arteriogenesis has yet to be delineated.

It is plausible that the Notch pathway interacts with other factors, such as CD36, to produce differential responses via regulation of VEGFR2 signaling. CD36 is a well-known anti-angiogenic receptor that is mainly expressed in microvascular ECs (MVECs) and arteriolar ECs in the vascular system. CD36 downregulation has been linked to the upregulation of pro-angiogenic and arterial genes (Ren et al., 2016). Several studies have demonstrated the association of CD36 expression with both angiogenesis and *de novo* arteriogenesis in adult organisms under oncogenic and pathological conditions (Ren et al., 2011, 2016; Dong et al., 2017). However, the relationship between CD36 and Notch signaling remains largely unknown. Jabs et al. (2018) demonstrated that in CD36 transcription is upregulated by molecules known to function downstream of Notch in vascular ECs. In addition, Notch signaling may play a significant role during arteriogenesis by interacting with such other factors as VEGF, FoxO1, and potentially CD36 (Ren et al., 2010, 2011, 2016; Ren, 2018). This potential interaction could be a *de novo* process, particularly under pathological conditions and occur within the TME (Ren et al., 2019).

### Notch Signaling in Endothelial Differentiation and Fate Determination

Regulation of cell fate decisions is a hallmark characteristic of Notch signaling. In the vasculature, a primary effect of Notch signaling is the promotion of stalk/tip cell specification (Mack and Iruela-Arispe, 2018). During angiogenesis, Notch signaling coordinates tip versus stalk phenotypes within a growing sprout. Expression of DLL4 at the end of the sprout (tip cell) activates Notch1 in the stalk cell (Blanco and Gerhardt, 2013).

Crosstalk between VEGF and Notch signaling is fundamental to sprout formation, vascular EC maintenance, and the establishment of EC heterogeneity (Blanco and Gerhardt, 2013). When Notch is inactivated at the onset of angiogenesis, there is a significant expansion of tip cells at the expense of stalk cells (Mack and Iruela-Arispe, 2018). Moreover, Notch inactivation can result in the loss of vascular hierarchy as tip cells are unable to organize tubes or form stable junctional complexes (Mack and Iruela-Arispe, 2018). Thus, Notch signaling is required for vascular stabilization and differentiation of the emerging vascular tree through the suppression of EC proliferation and stabilization of cell–cell junctions.

Notch signaling also plays a role to prevent excessive sprouting. Upon DLL4-mediated Notch activation, the induction of tip cells is repressed by a process called “lateral inhibition.” During lateral inhibition, VEGF signaling promotes the upregulation of DLL4 in tip cells, which in turn trans-activates Notch in the neighboring cells. Interestingly, a recent mathematical model of Notch-Jagged signaling revealed intriguing aspects about the existence of a third EC state. In addition to the two EC states (Tip or Stalk), Boareto et al. (2015) reported a novel hybrid state that exhibits intermediary tip and stalk cell characteristics, which may result from the asymmetric effects of the Notch intracellular domain. This allows neighboring cells to attain either opposite or similar fates, depending on which ligand is dominant (Delta-mediated signaling drives neighboring cells to have an opposite fate versus Jagged-mediated signaling drives the cell to maintain a similar fate). Additionally, Venkatraman et al. (2016) further confirmed the presence of a hybrid state apart from the traditional tip and stalk states. They showed that EC patterning dynamics does not solely depend upon the presence of Jag and this hybrid state can be found in both normal and pathological conditions. More evidence was provided by Koon et al. (2018), who, in addition to providing a computational model, also demonstrated the presence of the hybrid state by immunofluorescent stains for the known tip cell markers Delta and CD34. Importantly, the canonical tip cells, stalk cells and hybrid cells can be distinguished based upon the fluorescent signal intensity and the position of cells.

**FIGURE 2:**
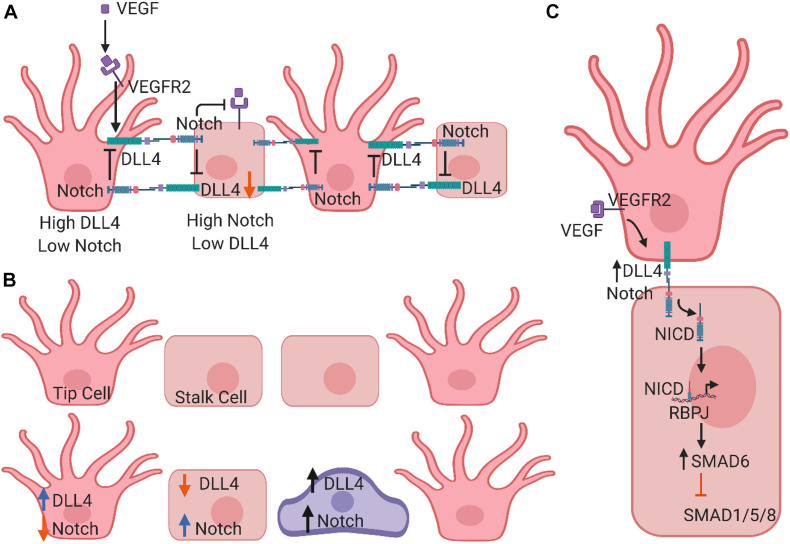
Schematic models of Notch signaling in vascular endothelial cell (EC) differentiation. **(A)** In endothelial tip cells, VEGF signaling induces DLL4 expression, which activates Notch1 signaling. In endothelial stalk cells, activation of Notch signaling via DLL4 suppresses differentiation toward a tip cell phenotype. **(B)** Graphic representation of lateral inhibition to show a classical salt and pepper pattern where tip cells are separated exactly by one stalk cell. DLL4 of one cell binds to the Notch receptor of the adjacent cell, and Notch inhibits DLL4 expression and the VEGFR2 signaling within the same cell. **(C)** Mathematic model of Notch signaling in asymmetric patterning. Cells do not necessarily adopt a canonical salt and pepper pattern. Instead, two tip cells might be separated by a few stalk cells, which could be determined by Jag. It is also possible that an intermediary tip cell may express similar DLL4 and Notch expression. Both theories involve a third EC state.

Notch signaling is also required for the proper formation of vascular branches. Recent studies have demonstrated that Notch regulates endothelial branching in response to other pathways, including BMP and SMAD1/5/8 signaling (Mack and Iruela-Arispe, 2018). SMAD6, an inhibitory protein that filters input signals from SMAD1/5/8, is regulated intrinsically by Notch levels (Mouillesseaux et al., 2016). The distribution of vascular sprouts depends upon the “Notch status” of a given cell within the sprout. Furthermore, the decision to form a new sprout or to widen the original vessel relies upon differential expression patterns of Notch-DLL4, BMP, and VEGF between cells (Mack and Iruela-Arispe, 2018) (Figure 2).

Lawson et al. (2001) showed in zebrafish embryos that the loss of Notch signaling leads to molecular defects in arterial venous differentiation. This finding might constitute an important link between blood flow and maintenance of Notch1 expression in adult arteries. We recently showed that a lipid signaling mediator lysophosphatidic acid (LPA) may promote angiogenesis and induce the arteriolar differentiation of vascular ECs via protein kinase PKD-1, a process in which the expression of DLL4 may play an important role (Ren et al., 2011, 2016; Best et al., 2018; Jiang et al., 2020). Acting via transcription factor FoxO1, PKD-1 could dictate arterial fate by the regulation of MVEC transdifferentiation (Ren et al., 2016; Ren, 2018). This may be associated with its regulation of CD36 transcription and fatty acid metabolism (Ren et al., 2016; Dong et al., 2017). Furthermore, Notch signaling may interact with the MAPK/Erk1/2 pathway, downstream of PKD-1 signaling, to promote arterial differentiation through inducing expression of ephrin B2, Hey2, and DLL4 during arteriogenesis under either developmental or ischemic conditions (Ren et al., 2010, 2016).

A recent study that combined computational modeling with *in vivo* experiments demonstrated that a VEGF-mediated positive signaling feedback can shape the timing, magnitude, and robustness of determining tip cell identity for angiogenic branching, which is terminated by DLL4-Notch signaling. Mechanistically, the induction of expression of tm4sf18 (an atypical tetraspanin) dictates the speed and robustness of EC selection for angiogenesis by stimulating VEGF signaling (Page et al., 2019). Intriguingly, this study demonstrates that Tm4sf18-mediated positive feedback appears to be specific to VEGF-induced arterial EC sprouting, implicating its functional role in *de novo* arteriogenesis.

On the other hand, Guarani et al. (2011) showed that NAD-dependent deacetylase sirtuin-1 (SIRT1) acts as an intrinsic negative modulator of Notch signaling in ECs. They also found that acetylation of the NICD on conserved lysine residues controls the amplitude and duration of Notch responses by altering NICD protein turnover. SIRT1 associates with NICD and functions as a NICD deacetylase, which opposes acetylation-induced NICD stabilization. Consequently, ECs lacking SIRT1 activity are sensitized to Notch signaling, resulting in impaired growth, sprout elongation, and enhanced Notch target gene expression in response to DLL4 stimulation, thereby adopting a non-sprouting, stalk-cell-like phenotype. *In vivo*, inactivation of SIRT1 in zebrafish and mice reduces vascular branching due to enhanced Notch signaling (Guarani et al., 2011).

### Notch Signaling in Proliferation and Apoptosis of Both Endothelial and Cancer Cells

The formation of a new blood vessel requires not only the selection of an endothelial tip cell, but also EC proliferation to enable sprout growth in length and diameter (Phng and Gerhardt, 2009). Notch signaling is required for the stabilization and differentiation of the emerging vascular trees, and it does so by suppressing EC proliferation and stabilization of cell–cell junctions. Several studies have shown that Notch signaling inhibits proliferation in ECs (Noseda et al., 2004; Liu G. et al., 2006; Harrington et al., 2008; Trindade et al., 2008). Notch signaling can inhibit the sprouting of ECs during the growth of blood vessels through the direct interaction between Notch receptors and DLL4 (Lobov and Mikhailova, 2018), further suggesting that DLL4 plays a fundamental role in vascular development and angiogenesis.

The Notch pathway also plays a significant role in apoptosis of both ECs and cancer cells. Notch appears to regulate apoptosis through extensive networks involving cell cycle, growth, and survival pathways. The Notch receptors seemingly play opposite roles in the endothelium. For instance, Notch1 and Notch4 prevent apoptosis induced by lipopolysaccharides (LPS) by increasing the levels of Bcl2 (Rizzo et al., 2013), TNF-α, and turbulent blood flow (Walshe et al., 2011). The activation of Notch signaling in human umbilical vein ECs (HUVECs) by estrogens promotes EC survival in response to TNF-α via signaling crosstalk between E2 and the Notch pathway (Fortini et al., 2017). This protective role may be attributable to Notch1-mediated Akt phosphorylation (Fortini et al., 2017) and estradiol-mediated protection via the estrogen receptor-β. On the other hand, Notch2 sensitizes ECs to apoptosis by inhibiting the expression of survivin, a key anti-apoptotic regulator (Quillard et al., 2009). Furthermore, during vascular remodeling, the activation of Notch signaling can cause EC apoptosis and thereby disrupt the balance of EC proliferation and regeneration (Dejana et al., 2017). Specifically, an increase of Notch3 can downregulate the expression of the anti-apoptotic gene *Bcl-1* in ECs, leading to apoptosis (Kartalaei et al., 2015; Dejana et al., 2017). The effects of Notch signaling on apoptosis has also been discussed by Aquila et al. (2019) in a recent review.

In the context of cancer, Notch signaling has been associated with multiple mechanisms of apoptosis. For example, the activation of Notch1 in colon cancer inhibits apoptosis by repressing p27 expression (Hristova et al., 2013; Meisel et al., 2020). Other studies have reported that the inhibition of Notch signaling by miRNAs leads to an increase in cancer cell apoptosis. For example, Du et al. (2017) showed that miR-145 induces glioma cell apoptosis by inhibiting Notch signaling through the binding of Bcl2/adenovirus E1b interacting protein 3 (BNIP3) to its 3′-UTR in glioma cells. Additionally, Li et al. (2018) demonstrated that overexpression of miR-22 promotes apoptosis in ovarian cancer cells by suppressing Notch signaling. Therefore, the Notch pathway could represent a promising therapeutic target in certain types of cancer since suppression of Notch signaling results in the loss of the malignant phenotype in both *in vitro* and *in vivo* models. However, the toxicity of Notch inhibitors to normal cells should be considered. To minimize the side effects, cancer-specific targeting should be an optimal strategy.

### Notch Signaling in Endothelial to Mesenchymal Transition

Endothelial-to-mesenchymal transition (EndoMT) is a process where ECs lose their endothelial-specific phenotype and progressively acquire mesenchymal properties such as formation of spindle-shaped elongated cell morphology, loss of cell–cell junctions and polarity, and increased cellular motility, loss of endothelial markers (such as CD31 or vascular endothelial (VE) cadherin), and the acquirement of mesenchymal features [such as expression of N-cadherin, fibroblast-specific protein 1 (FSP1), or α-smooth muscle actin (αSMA)] (Zeisberg et al., 2007a; Azhar et al., 2009).

**FIGURE 3:**
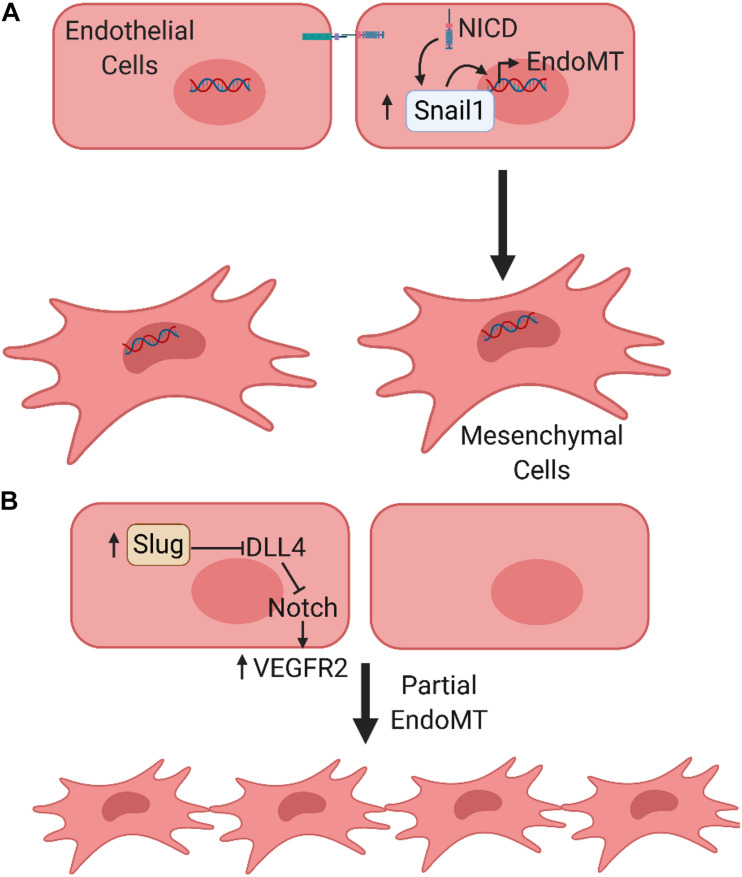
Notch signaling regulates endothelial to mesenchymal transition. **(A)** Notch signaling induces an endothelial to mesenchymal transition (EndoMT). As an example, active NICD regulates the expression of Snail1, a key EndoMT transcription factor to promote EndoMT. **(B)** Partial EndoMT, where cells temporarily lose apical-basal polarity and gain migratory capacity, while retaining intercellular junctions and undergoing collective rather than individualized cell migration.

As major components of the vascular networks in the TME, ECs demonstrate plasticity and the potential to transdifferentiate into mesenchymal cells via EndoMT. EndoMT has been shown to participate in critical embryonic developmental processes and the pathogenesis in a variety of genetic and acquired human diseases including cancer-associated fibrosis (Zeisberg et al., 2007a), cardiac fibrosis (Zeisberg et al., 2007b), and atherosclerosis (Chen P.Y. et al., 2015). A recent review highlighted important roles of EndoMT within the TME during tumor progression and the acquisition of therapeutic resistance (Clere et al., 2020). Cells that underwent EndoMT increased expression of the Snail family proteins, which plays a critical role in disrupting cell–cell junctions (Potenta et al., 2008). Furthermore, Notch can either directly induce or suppress gene expression. As an example, Notch signaling can upregulate Snail and Slug to ultimately initiate EndoMT in developmental and pathological conditions (Niessen et al., 2008; Welch-Reardon et al., 2015) (Figure 3).

In addition to the complete epithelial-to-mesenchymal transition (EMT)/EndoMT, the concept of partial EMT/EndoMT has emerged recently, which was described by Welch-Reardon et al. (2015). Examples of a partial EndoMT process include angiogenic sprouting, the crosstalk between Notch and VEGF pathways in hypoxic tumors to form unstable and leaky vessels (Holderfield and Hughes, 2008), and a process involving tubulogenesis (cells temporarily lose apical-basal polarity and gain migratory capacity, but never fully acquire all mesenchymal phenotypes) (Welch-Reardon et al., 2015). However, the molecular signaling and mechanisms by which epithelial or endothelial cells undergo either complete or partial EMT/EndoMT are largely unknown. Welch-Reardon et al. (2015) proposed that Notch signaling, being a contact-dependent pathway, may play an important role in the case of sprouting angiogenesis (Welch-Reardon et al., 2015). Their recent study demonstrated that endothelial Slug activity may be involved in driving the transition of ECs toward a partial EndoMT state. This activity promotes sprouting angiogenesis by modulation of Notch signaling via transcriptional repression of DLL4 and upregulation of VEGFR2 (Hultgren et al., 2020). It should also be noted that anti-angiogenic signaling could be involved in the regulation of arterial sprouting and angiogenesis under complex physiological conditions *in vivo*, particularly under pathological conditions such as tumor angiogenesis (Ren et al., 2006, 2009, 2016; Kazerounian et al., 2011). The association between this process, EndoMT and the Notch pathway warrants further exploration.

Notch activation in conjunction with sprouting angiogenesis could be a crucial determinant of either partial or full EndoMT. However, other signaling pathways such as Wnt and TGF-β signaling pathway are also capable of promoting partial EndoMT. Mechanistic studies have demonstrated a role for both TGF-β and bone morphogenic protein (BMP) in conjunction with Notch signaling to regulate EndoMT in embryonic heart formation (Thiery and Sleeman, 2006). In addition, the role of Notch signaling in EndoMT is prominent during heart valve development, arterial venous differentiation, and the remodeling of the primitive vascular plexus (Lin et al., 2012). Intriguingly, inhibition of Notch signaling abrogates TGF-β-stimulated EndoMT in HUVECs (Chen X. et al., 2015).

While the interaction between Notch and EndoMT is pivotal during tumor progression via a complex modulation of both the tumor and the surrounding stroma (Nie et al., 2014). Ghiabi et al. (2015) showed that xenograft cells from two different breast cancer cell lines, MDA-MB-231 and MCF-7, promoted the acquisition of a contact-dependent mesenchymal phenotype in ECs that contributed to the generation of a pro-tumoral niche. The authors concluded that Notch signaling likely induces a tumor-fostered mesenchymal phenotype in ECs, while the endothelial characteristics of ECs remain unchanged (the cells maintain EC traits). This suggests that Notch signaling might promote partial EndoMT. In the context of CSCs, Marín-Ramos et al. (2019) demonstrated that Notch signaling is necessary for TGF-β-stimulated EndoMT in glioblastoma.

These studies strongly support a role for Notch signaling in the development of multiple biological processes and diseases through regulation of EndoMT. Significantly, it is worth investigating whether and how Notch signaling contributes to the development of metastatic niche in tumor progression and therapeutic resistance through promoting EndoMT and/or partial EndoMT, either of which might play a critical role in the regulation of TME plasticity and CSC heterogeneity.

## Notch Signaling in Tumor Progression

### Overview

Dysregulated Notch signaling has been documented in a variety of human cancers including melanoma (Ntziachristos et al., 2014), lymphoma (Aster et al., 2017), glioma (Teodorczyk and Schmidt, 2014), breast cancer (Guo et al., 2011), ovarian cancer (Ntziachristos et al., 2014), head and neck cancer (Ntziachristos et al., 2014), colorectal cancer (Ntziachristos et al., 2014), pancreatic adenocarcinoma (Ntziachristos et al., 2014), hepatocellular carcinoma (Ntziachristos et al., 2014), glioblastoma (Ntziachristos et al., 2014), thyroid cancer (Guenter et al., 2021), and neuroendocrine cancer (Ren et al., 2019). Given the involvement of Notch signaling in many different cancers, this pathway has become a key target for developing therapeutics to treat broad spectrum of patients. However, the role of Notch signaling in tumorigenesis often depends upon the type of cancer. Depending upon the context, Notch activation can be either oncogenic or tumor suppressive. The opposing roles of Notch signaling may be attributed to the innate function of Notch in a cell type-dependent manner, and to crosstalk between Notch signaling and other cellular pathways.

Oncogenic Notch signaling activation is often linked to an increase in cellular proliferation, inhibition of apoptosis, suppression of cellular differentiation, and the induction of CSC features (Aster et al., 2017; Brzozowa-Zasada et al., 2017). In T-cell acute lymphoblastic leukemia (ALL), a mutation that causes constitutive activation of Notch1 drives tumorigenesis by regulating Notch-dependent transcription factors that promote cell growth, proliferation, and self-renewal (Weng et al., 2004; Sanchez-Martin and Ferrando, 2017). In ovarian cancer, aberrant activation of Notch signaling contributes to chemoresistance, while overexpression of the Notch3 receptor correlates with advanced stage disease, metastasis, and poor overall survival (Jung et al., 2010; Brzozowa-Zasada et al., 2017).

The tumor suppressive role of Notch has been described in other malignancies including bladder squamous cell carcinoma (bladder SCC), medullary thyroid cancer (MTC), and skin cancer (Weng and Aster, 2004; Dotto, 2008; Rampias et al., 2014; Jaskula-Sztul et al., 2015; Tamagnone et al., 2018). In bladder SCC, mutations that ablate Notch activity lead to an earlier onset of disease and more aggressive tumor progression (Nowell and Radtke, 2017). In MTC, overexpression of Notch1 and Notch3 may limit tumor cell growth and proliferation *in vitro* and *in vivo* in a dose-dependent manner (Jaskula-Sztul et al., 2011, 2015). Similarly, mice that conditionally lack Notch1 in keratinocytes demonstrate increased keratinocyte proliferation and delayed terminal differentiation, thereby leading to increased susceptibility of skin cancer formation (Rangarajan et al., 2001; Weng and Aster, 2004).

The crosstalk between Notch and other cell signaling pathways may explain some of the contrasting tumorigenic consequences that are observed in different types of cancer. Notch signaling is known to interact with multiple other oncogenic signaling pathways such as epidermal growth factor receptor (EGFR), platelet-derived growth factor (PDGF), NF-κB, Akt, Hedgehog, mTOR, Ras, Wnt, estrogen receptor (ER), and androgen receptor (AR) signaling (Guo et al., 2011; Baker et al., 2014; Jeon et al., 2014; Qiu et al., 2014; Meurette and Mehlen, 2018; Hibdon et al., 2019; Katoh and Katoh, 2020).

The interaction of Notch1 with NFκB promotes tumor progression in colorectal adenocarcinoma through inhibition of apoptosis via the upregulation of the Bcl-xL (Gopalakrishnan et al., 2014). The communication between Notch and EGFR signaling may lead to more aggressive disease due to acquired resistance to EGFR-targeted chemotherapeutics through enhanced EMT characteristics (Xie et al., 2013), a behavior often associated with cancer aggressiveness. Notch signaling likely contributes to EMT through Notch1-mediated down-regulation of E-cadherin (epithelial marker) and up-regulation of Vimentin and Snail (mesenchymal markers) (Yuan et al., 2014; Kar et al., 2019).

Conversely, Notch1 functions as a tumor suppressor to transform high-grade adenomas into low-grade ones in murine models of colon cancer by epigenetically suppressing Wnt pathway target genes (Kim et al., 2012). In addition, pharmacologic activation of both Notch1 and Raf-1 signaling in MTC cells reduces aberrant secretion of hormones and tumor cell proliferation, suggesting a tumor suppressive role for Notch1 signaling in this context.

To better understand the role of Notch signaling in tumor development, it is important to consider the innate function of Notch in the cell or tissue type being studied. Just as the impact of Notch signaling varies among cancer types, it also varies among normal tissue types. For example, Notch signaling plays different roles during the normal development of different cell types in the pancreas. The Notch pathway plays a pivotal role in deciding the fate of progenitor cells as either exocrine cells (acinar/ductal cells) or endocrine cells (Islets of Langerhans) by lateral inhibition (Hald et al., 2003; Ahnfelt-Rønne et al., 2007; Kim et al., 2010; de Back et al., 2013; Pézeron et al., 2014; Li et al., 2016).

In summary, Notch signaling has a significant functional role in a broad spectrum of human cancers. The oncogenic involvement of Notch signaling across different cancer types has emphasized its centralized role in the regulation of tumor formation, progression, and therapeutic resistance. The distinct role of Notch signaling in tumor progression emphasizes the need for further studies to better understand this pathway in a cellular- and tumor type-dependent manner. A deeper understanding of Notch signaling in various biological settings and pathological conditions may reveal key opportunities for effective therapies that target Notch in tumors. We will discuss the role of Notch signaling in tumor angiogenesis, cancer stem cells (CSCs), therapeutic resistance, and signaling crosstalk between CSCs and vascular ECs as well as the clinical potential of targeting the Notch signaling pathway in more detail.

### Notch Signaling in Tumor Angiogenesis

Tumor angiogenesis is characterized by excessive endothelial sprouting from preexisting blood vessels, which leads to overgrowth of randomly organized tumor vessels including venous and arteriolar vessels and capillaries (Sitohy et al., 2011; Nagy and Dvorak, 2012; De Palma et al., 2017; Ren et al., 2019; Yunus et al., 2019). The growth of new vascular networks or blood vessels within the TME is pivotal during tumor growth and progression, as newly formed and proliferating cancer cells require a supply of oxygen and nutrients to survive and remove waste products (Carmeliet, 2005; Jain and Carmeliet, 2012). They may also serve as a favorable niche for CSCs and as a channel system for both metastatic CSCs and the infiltration of immune cells.

Stimulation of neoangiogenesis in the TME depends upon the induction of such pro-angiogenic factors as VEGF and basic fibroblast growth factor (bFGF) (Yancopoulos et al., 2000; Lieu et al., 2011; Lugano et al., 2020). The crosstalk between pro-angiogenic and anti-angiogenic factors may determine angiogenic status throughout tumor progression (Ren, 2016), in which angiogenic receptors play a critical role in mediating angiogenic signaling. In fact, there is a *Yin and Yang* balance between pro-angiogenic and anti-angiogenic signaling to control overall angiogenic responses. During this dynamic angiogenic process, angiogenic receptors may serve as a pivotal axis to regulate the angiogenic functions of corresponding ligands. As an example, VEGFR2 is critical for upregulating VEGF signaling, while CD36 signaling may function as a critical negative regulator of angiogenesis (Ren, 2016).

Several studies have highlighted the critical role of Notch signaling in the overall regulation of tumor angiogenesis (Siekmann and Lawson, 2007; Suchting et al., 2007). The Notch ligands DLL4 and Jag1 are the best characterized Notch pathway factors that have roles in tumor angiogenesis. DLL4 is highly expressed in tumor ECs and its expression can be regulated by VEGF, bFGF, and by low oxygen conditions (hypoxia) through hypoxia-inducible factor 1α (HIF1α) (Dufraine et al., 2008). The importance of DLL4 in tumor angiogenic processes is highlighted by the fact that silencing its expression leads to an inhibition of proliferation and migration of ECs (Patel et al., 2005; Rehman and Wang, 2006).

Intriguingly, tumor vasculature becomes disorganized and overgrown upon pharmacologic inhibition of DLL4, ultimately reducing overall tumor growth due to dysfunctional tumor angiogenesis (Dufraine et al., 2008). Given the ability of DLL4 to regulate tumor angiogenesis, strategies that target Notch/DLL4 signaling may show therapeutic potential against cancers. Unlike DLL4, Jag1 is overexpressed in cancer cells. It is suspected that Jag1 serves as a communication factor between cancer cells and tumor-associated ECs (TECs) to activate Notch signaling and induce angiogenesis by increasing cell proliferation and stabilizing vessels (Dufraine et al., 2008).

Notch signaling also regulates tumor angiogenesis and metastasis in a context-dependent manner. Notch signaling can upregulate Jag1 to antagonize the DLL4-dependent ‘stalk’ phenotype, thereby promoting EC sprouting and proliferation (Bridges et al., 2011; Simons et al., 2016). Activation of Notch signaling in ECs may either promote or inhibit metastasis in the lung (Wieland et al., 2017) and liver (Wohlfeil et al., 2019), respectively. Sustained endothelial Notch1 signaling facilitates metastasis by generating a senescent, pro-inflammatory endothelium in human carcinomas and melanoma (Wieland et al., 2017). Intriguingly, Notch activation in liver ECs protects hepatic metastasis by regulating endothelial-tumor cell adhesion independent of angiocrine signaling in malignant melanoma and colorectal carcinoma (Wohlfeil et al., 2019). These studies indicate the complex roles of the Notch pathway in tumor progression. Further investigation into the mechanisms by which Notch signaling regulates both cancer cells and TECs within the TME is warranted for the discovery of novel and effective strategies to control tumor progression.

### Notch Signaling in Cancer Stem Cells (CSCs)

Accumulating evidence in CSC research suggests that the principles of anti-cancer treatments should be revised with consideration for the intrinsic heterogeneity of cancer cells. CSCs are a subpopulation of cells within a tumor that are able to promote neoplastic initiation, growth, survival, and metastasis (Al-Hajj et al., 2003; Li et al., 2007; Zhang et al., 2008). CSCs are hierarchically organized cancer cell populations within tumors and can also be defined by their ability to self-renew, exclusively retain tumorigenic potential, and generate heterogeneous lineages of cancer cells. Additionally, CSCs are resistant to a variety of conventional chemotherapies and radiotherapies, which leads to the failures of most conventional cancer therapies (Kim et al., 2009).

Notch signaling has emerged as a key pathway in CSCs. This signaling pathway may be involved in the regulation of survival, plasticity, and self-renewal of CSCs. Blockade of the Notch pathway using γ-secretase inhibitors can decrease CSC numbers, increase apoptosis, and limit tumorsphere formation efficiency, a measure of cancer stemness (Abel et al., 2014). However, the precise mechanisms by which Notch signaling promotes and maintains CSCs are still being investigated.

The expression of both Notch receptors and ligands have been correlated with CSC-like phenotypes in a variety of cancers, with a number of studies in breast cancers (Bray, 2006; Xiao et al., 2017; Yang et al., 2020). Breast cancer cell lines contain functional CSCs with distinct molecular signatures and metastatic capacities (Charafe-Jauffret et al., 2009). Aberrant Notch signaling also plays an important role in the invasive properties of breast CSCs (Dontu et al., 2004; Sansone et al., 2007; Guo et al., 2011). Higher Notch1 expression is associated with a transition from ductal carcinoma *in situ* to invasive cancer and may worsen overall and recurrence-free survival (Yuan et al., 2015). In particular, the activation of Notch3 through an IL-6 driven feed forward loop is able to promote self-renewal of breast CSCs (Sansone et al., 2016). In addition, the activation of Notch signaling may enhance the CSC-like properties of breast cancer cells likely via interaction with and the activation of LPA/PKD-1 signaling axis (Bray, 2006; Jiang et al., 2020).

Furthermore, the interaction of Notch1 with the erythropoietin receptor (EpoR), a protein expressed on the surface of breast CSCs, can lead to the maintenance of stemness through continuous self-renewal (Phillips et al., 2007). Notch1 also promotes the stem cell phenotype via activation of c-Jun signaling in a triple negative breast cancer (TNBC) (Xie et al., 2017). It has been speculated that the mechanism by which Notch promotes stemness is through activation of NF−κB as a means of resisting apoptosis (Baker et al., 2018; Najafi et al., 2019). A recent study demonstrates that the serine-threonine kinase receptor associated protein (STRAP) is oncogenic and regulates stemness behavior and drug response in colorectal cancer via mediating the epigenetic activation of Notch signaling (Jin et al., 2017).

Finally, it should be noted that heterogeneous CSC subsets in primary tumors have varied EMT phenotypes, in which Notch signaling may play a critical role. A recent study demonstrated that Notch-Jag1 signaling triggered by inflammatory cytokines can lead to varying EMT phenotypes within the TME (Bocci et al., 2019). The authors of this study also showed that breast CSCs with a mesenchymal-like (CD44^+^/CD24^–^) phenotype reside at the invasive edge of the tumor, whereas a population of hybrid epithelial-mesenchymal (E/M) CSCs are localized within the tumor interior. Thus, a primary tumor may contain spatially distributed cells with varying extents of EMT. This spatial distribution of CSCs and EMT heterogeneity may be attributed to a TGF-β diffusion gradient and cell–cell communication through Notch-Jag1 signaling induced by inflammatory cytokines such as IL-6. Furthermore, knockdown of Jag1 in the hybrid E/M SUM149 TNBC cell line suggests a role for Notch-Jag1 signaling in the maintenance of the CSC phenotype (Bocci et al., 2019). Despite the limitation of modeling itself and associated experimental evidence, this study provides insight into a concept regarding the spatiotemporal patterning of CSC subsets in the TME in response to Notch signaling, which may occur in a variety of other cancer types.

### Notch Signaling in Cancer Therapeutic Resistance

The Notch signaling pathway may be a key factor in the context of anti-cancer drug resistance (Wang et al., 2010; Zou et al., 2013). Specifically, the activation of Jag1/Notch1 signaling has been associated with chemoresistance in many human tumors, including ovarian cancer (Liu et al., 2014), and the activation of the Notch1 signaling induces resistance to cisplatin (Liu et al., 2013; Wang et al., 2014; Zeng et al., 2020). Studies that investigate Notch in the context of drug resistance emphasize that Notch signaling can regulate the maintenance and self-renewal of CSCs and induce the EMT phenotype, both features associated with drug resistance and metastasis (Liu et al., 2013; Wang et al., 2014; Zeng et al., 2020).

Different chemotherapeutic agents can upregulate the expression of CSC-related markers and stimulate the expansion of CSCs (Phi et al., 2018; Yang et al., 2020). A recent study reported a significant increase in the percentage of CD44^+^/CD24^–^/low breast CSCs in biopsies specimens following chemotherapy (Abraham et al., 2005), suggesting that modulating Notch in breast cancers may be a promising strategy to reduce drug resistance by eliminating the CSC subpopulation (Shah et al., 2018; Najafi et al., 2019). In glioblastoma, CD133^+^ CSCs are resistant to several chemotherapeutic agents (Liu Z.J. et al., 2006).

Additionally, CSCs are more resistant to radiation than non-CSCs (Rycaj and Tang, 2014; Arnold et al., 2020). Radiation can increase the percentage of CD133^+^ CSCs *in vitro* and *in vivo* (Liu Z.J. et al., 2006; Kawamoto et al., 2012). This phenomenon was also seen in breast cancers, where tumors were found to be enriched for the CD44^+^/CD24^–^ CSC subpopulation following exposure to radiation (Gao et al., 2016). In these CSC-enriched subpopulations, Notch signaling was preferentially activated in response to radiation exposure, suggesting its possible role for this pathway in radiation resistance of breast CSCs (Gao et al., 2016). This is supported by multiple studies showing that CSCs activity is regulated by Notch signaling (Kanwar et al., 2010; Gao et al., 2016; Yang et al., 2020).

Blocking Notch signaling in tumors could reduce drug resistance and CSC properties, potentially leading to increased effectiveness of chemotherapeutics. This concept is beginning to be explored pre-clinically and has shown promising early results. In xenograft models of pancreatic cancer, the combination of a small molecule Notch inhibitor with gemcitabine reduced tumor proliferation and angiogenesis, leading to an overall reduction in tumor growth and metastasis (Yabuuchi et al., 2013; Zou et al., 2013).

In summary, Notch signaling appears to play a role in cancer drug resistance, potentially by regulating the CSC subpopulation. Therefore, targeting Notch signaling may demonstrate therapeutic potential for cancers by overcoming drug resistance. This may be accomplished by eliminating CSCs and/or EMT-type cells that are typically believed to be the “root cause” of tumor recurrence and metastasis. To develop more effective therapies, it is essential to have a better understanding of the molecular mechanisms by which Notch signaling mediates the CSC phenotype in certain cancers to promote drug resistance. Additional studies are also necessary to understand the potential clinical impact of using Notch inhibition strategy to target CSCs in either a single or combination treatment regimen.

### Notch Signaling Crosstalk Between Cancer Stem Cells and Vascular Endothelial Cells

The crosstalk between tumor cells and their microenvironment is crucial for cancer cell self-renewal, tumor growth, and metastasis (Butler et al., 2010; Hanahan and Weinberg, 2011). Notch pathway activation by signals within the TME may be an additional mechanism by which Notch controls the activity of CSCs (Ingangi et al., 2019). In colorectal cancer, Notch signaling may not be essential for bulk tumorigenesis, but signaling crosstalk between Notch1 and FGF/WNT pathway could be responsible for orchestrating the TME for CSC maintenance (Germann et al., 2014; Mourao et al., 2019). This concept is supported by the fact that Notch signaling mediates cell–cell communication (Lai, 2004; Stylianou et al., 2006).

A major component of the TME is the vascular niche, which is composed of a vascular basement, pericytes (capillaries), and smooth muscle cells (arteriolar and venous vessels) surrounding ECs (Ping et al., 2016; Ingangi et al., 2019). A seminal study along with our recent work have demonstrated that some aggressive cancer cells may prefer high perfusion that provides nutrients and oxygen rather than hypoxia conditions as CSCs and aggressive cancer cells tend to localize themselves near blood vessels and arterioles within the TME (Kumar et al., 2019; Jiang et al., 2020).

Vascular ECs are an essential component of blood-vessel walls, which are the main driver of new blood vessel formation including initiation of vessel sprouting and vessel maturation. Notch signaling in vascular ECs may promote the stem cell phenotype including self-renewal of CSCs and tumor progression (Zhu et al., 2011; Lu et al., 2013; Cao et al., 2014, 2017; Ghiabi et al., 2014). It has been suggested that ECs regulate the homeostasis of CSCs directly via cellular contact or indirectly by releasing a distant specific growth factor called stem cell-active trophogens (Plaks et al., 2015; Ingangi et al., 2019; Jiang et al., 2020). For example, ECs in TNBC can enhance self-renewal, survival, and pro-metastatic properties of the breast CSCs through direct cell–cell contact mediated by Notch signaling *in vitro* (Ghiabi et al., 2014).

In glioblastoma, Notch signaling can induce CSC features through bFGF and regulate the EC-mediated acquisition of CSC properties (Fessler et al., 2015; Bradshaw et al., 2016; Bazzoni and Bentivegna, 2019; Jiang et al., 2020). Inhibition of Notch signaling can suppress the self-renewal of glioblastoma CSCs by reducing the number of ECs (Bradshaw et al., 2016). In fact, co-culture of ECs with glioblastoma CSCs increases the self-renewal capability and maintains an undifferentiated status of the CSCs through direct cell–cell contact and indirect paracrine signaling involving DLL4/Notch (Nandhu et al., 2014; Rostama et al., 2014).

Beyond the self-renewal potential and the capacity to generate heterogeneous cancer cell populations, CSCs can transdifferentiate into ECs and develop vascular mimicry (Jain and Carmeliet, 2012; Huang et al., 2015). CSCs in glioblastoma, breast cancer, and ovarian cancer are able to differentiate into ECs (Jain and Carmeliet, 2012; Fan et al., 2013; Qian and Rankin, 2019). They also participate in vascular mimicry under hypoxic conditions (Fan et al., 2013; Qian and Rankin, 2019). Hypoxia is believed to play an important role in the CSC-mediated production of angiogenic factors and the maintenance of “stemness” within the CSC subpopulation (Qian and Rankin, 2019). The interaction between Notch signaling and HIF1α under hypoxic conditions (Qiang et al., 2012; De Francesco et al., 2018) suggests a potential role of Notch pathway in the development of vascular mimicry within the TME. Intriguingly, Notch signaling may also promote the development of the arteriolar niche within the TME that can maintain CSC features during tumor progression (Jiang et al., 2020).

In conclusion, the Notch pathway may determine the fate of both ECs and CSCs and mediate crosstalk between these two cell types. However, several studies indicate that the differentiation of CSCs is not a one-way route, but instead might be a reversible process that can be directed by signals from the TME (Huang et al., 2015). Furthermore, a recent study suggested that CSCs can differentiate to not only ECs but also tumor cells themselves, indicating the potential to generate endothelial phenotypes and directly participate in tumor angiogenesis and cancer cell plasticity (Dudley, 2012; Hida et al., 2018). Further studies are necessary to fully understand how the Notch pathway in the TME participates in the crosstalk between CSCs and ECs and when and how CSCs differentiate into ECs and/or tumor cells.

### Clinical Potential of Inhibiting Notch Signaling

Given the critical roles of Notch signaling in endothelial cell biology, angiogenesis, and the expansion of CSCs in a variety of tumors, targeting the Notch pathway holds great therapeutic potential. Unfortunately, there are no approved drugs that target this pathway for cancer treatment in the clinic. A recent review has discussed the therapeutic implications of cardiovascular disease and cardiotoxicity caused by anti-cancer drugs (Aquila et al., 2019). Our recent review also provides an update on the use of gamma secretase inhibitors (GSIs) to inhibit Notch signaling for cancer treatment (McCaw et al., 2020). However, much remains to be ascertained before optimal approaches targeting Notch signaling can be developed to treat patients with cardiovascular diseases and cancers.

One approach to inhibit the Notch pathway is through suppression of the proteolytic step that leads to the generation of the active Notch intracellular domain (NICD). Gamma-secretase is the orchestrating enzyme of the crucial proteolytic activity that releases the NICD and is a key player in the canonical Notch signaling pathway. GSIs prevent cleavage of the NICD by blocking the proteolytic function of presenilin enzymes, thereby preventing Notch pathway activation (Cook et al., 2018). Therefore, GSIs can inactivate Notch signaling in cancer cells and lead to impaired cancer cell growth and tumor progression. GSIs may also suppress angiogenesis in solid tumors by interfering in the crosstalk between the tumor and the vasculature (McCaw et al., 2020). Several different forms of GSIs have been tested for antitumor effects (Shih and Wang, 2007; McCaw et al., 2020). Phase I clinical trials using Notch inhibitors (MK0752, PF-03084014, RO4929097) either alone or in combination with other agents (Cook et al., 2018; McCaw et al., 2020) have been conducted in advanced breast cancers, lung cancer, melanoma. These studies strongly suggest a potential clinical application for GSIs in cancer therapeutics. However, the side effects associated with GSIs pose a major clinical challenge, since γ-secretase does not exclusively target Notch signaling in cancer cells alone. In addition to GSIs, a bi-specific antibody (ABL001) that targets both VEGF and DLL4 is currently being evaluated in a phase I clinical trial. This trial aims to assess the safety and tolerability of the bi-specific antibody in patients who did not respond to current chemotherapy or targeted therapy (Yeom et al., 2021).

Furthermore, inhibition of Notch signaling has shown efficacy in reducing CSC features, thereby limiting tumor progression *in vivo*. In murine models of colorectal cancer, blocking Notch signaling with an anti-DLL4 antibody reduced the frequency of CSCs, delayed recurrence of tumors, and decreased metastasis (Hoey et al., 2009; Najafi et al., 2019). Inhibition of Notch signaling by GSIs abolished the formation of secondary mammospheres from a variety of human breast cancer cell lines and patient samples (Grudzien et al., 2010), leading to the observation that Notch inhibition could overcome treatment resistance (McCaw et al., 2020). In an animal breast cancer model, GSIs alone do not reduce tumor volume. Comparatively, trastuzumab (HER2-targeted therapy) alone does indeed drastically decrease tumor volume but does not prevent recurrence. However, combining GSIs with trastuzumab stops the recurrence of HER2-positive tumors in mice (Pandya et al., 2011). The results of this study indicate that Notch inhibition through GSIs targets CSCs, while trastuzumab targets the tumor bulk. In combination, these two targeted mechanisms can eliminate the tumor and prevent recurrence. This approach may be attributed to the evidence that Notch signaling is essential for the maintenance of CSCs and the development of the arteriolar niche to promote the self-renewal of CSCs (Shutter et al., 2000; Lawson et al., 2001; Liu et al., 2003; Trindade et al., 2008; Jiang et al., 2020). Since Notch signaling is crucial in many biological processes, targeting alternative pathways that are critical for Notch signaling-mediated pathology could be tested as potential strategies for cancer treatment.

## Summary and Perspectives

**FIGURE 4:**
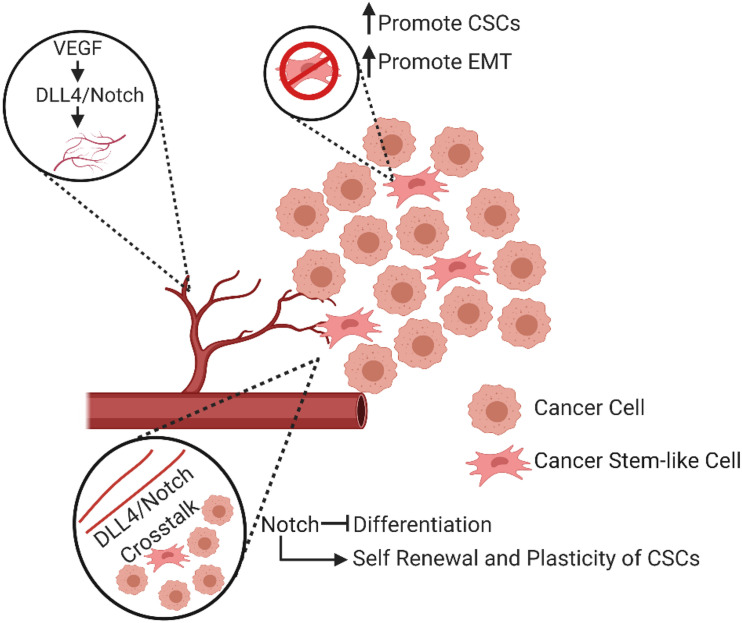
Diverse involvement of Notch signaling in angiogenesis and CSCs. The interaction between VEGF and DLL4/Notch signaling regulates angiogenesis. In addition, active Notch signaling can promote the development of the CSC phenotype, the renewal and plasticity of CSCs, and promote an epithelial to mesenchymal transition (EMT).

In many human cancers, the activation of Notch signaling can crosstalk with numerous oncogenic signaling pathways, such as Wnt and Hedgehog signaling, cytokines, and oncogenic kinases. This crosstalk plays an important role in tumor angiogenesis and growth, invasion, metastasis, and therapeutic resistance. As a major signaling pathway in tumor progression, Notch has been extensively studied in the context of angiogenesis and CSC biology. In vascular ECs, this pathway is essential for the determination of cellular fate, proliferation, apoptosis and EndoMT. Importantly, Notch signaling plays an essential role in arteriolar differentiation and the determination of angiogenic phenotype as well as in tumor initiation, EMT-driven metastatic growth, and the self-renewal of CSCs (Ranganathan et al., 2011). Furthermore, this signaling may contribute to CSC-mediated chemoresistance in many different types of cancer (Zhang et al., 2008; Meng et al., 2009; Ulasov et al., 2011; McAuliffe et al., 2012) and radiation resistance (Gao et al., 2016). In summary, Notch pathway activation is associated with more aggressive cancer via the regulation of angiogenesis and CSC features in a variety of tumors (Figure 4).

However, it should be noted that Notch signaling is characterized by its context-dependent consequences, exerting either tumor-suppressive or oncogenic effects. Using a reverse genetic CRISPR screen to functionally assess about 500 long tail gene mutations that occur in human head and neck squamous cell carcinoma (HNSCC), Loganathan et al. (2020) recently identified 15 tumor-suppressor genes with activities that converged on the Notch signaling pathway. This suggests that Notch inactivation drives HNSCC growth given that Notch itself is mutated at a high frequency in this type of cancer. In addition, Bill et al. (2020) demonstrated that EGF-like domain 7 (EGFL7), a secreted protein that plays an important role in acute myeloid leukemia (AML), contributes to Notch silencing in AML by antagonizing canonical Notch ligand binding. However, whether oncogenic or tumor suppressive, targeting Notch signaling holds promising therapeutic potential for cancer treatment.

Due to its driving role in many types of cancer, significant attention has been paid in recent years toward the development of clinically valuable antagonists of Notch signaling. However, there are still many gaps to uncover in this field due to the heterogeneity and complexity of the molecular biology in human cancers including neuroendocrine tumors and breast cancers as well as in cardiovascular diseases. In terms of cancer treatment, a popular new strategy is the development of target-selective “smart” drugs based upon the foundation of characterized molecular mechanisms. The crosstalk between Notch signaling and many other oncogenic signaling pathways in carcinogenesis presents multiple opportunities for the design of new drugs aimed at blocking these interactions. However, this would require new studies to better understand the precise mechanisms by which Notch signaling pathway and molecular signaling critical to the Notch pathway promotes or inhibits tumor progression. In addition, developing innovative tools to more accurately predict their prognosis, and designing and testing personalized treatment strategies would be helpful to facilitate the optimal Notch targeting in cancer patients.

Research in the past decade has focused upon eliminating tumor recurrence by targeting CSCs. Targeting Notch may be an optimal therapeutic strategy to disrupt the arteriolar niche and eradicate CSCs. However, targeting the Notch pathway may elicit cardiovascular toxicities because of its critical role in the maintenance of cardiovascular homeostasis by EC DLL4-Notch signaling (Potente et al., 2011; Goel and Mercurio, 2013). Through molecular profiling and screening, potential co-targeting options could be found to use Notch inhibition as a strategy to benefit patients (Mollen et al., 2018). Additional research will be required to determine the cellular and molecular mechanisms, as well as the precise role of Notch in different cancer types. This will enable deep investigations into the use of Notch-targeting agents and their combination with current therapeutic regimens to achieve optimal treatment efficacy.

## Author Contributions

AA, AG-G, and RG wrote the manuscript. AG-G made the figures. JR, AB, and HC reviewed the manuscript. BR conceptualized and wrote the manuscript. All authors approved the submitted version.

## Conflict of Interest

The authors declare that the research was conducted in the absence of any commercial or financial relationships that could be construed as a potential conflict of interest.
